# Real World Lab Data: Patterns of Lymphocyte Counts in Fingolimod Treated Patients

**DOI:** 10.3389/fimmu.2018.02669

**Published:** 2018-11-20

**Authors:** Maxi Kaufmann, Rocco Haase, Undine Proschmann, Tjalf Ziemssen, Katja Akgün

**Affiliations:** MS Center, Center of Clinical Neuroscience, University Hospital Carl Gustav Carus, University of Technology Dresden, Dresden, Germany

**Keywords:** fingolimod, lymphopenia, lymphocyte subsets, real world lab data, monitoring

## Abstract

**Objective:**

Fingolimod is approved for the treatment of highly active relapsing remitting multiple sclerosis (MS) patients and acts by its unique mechanism of action via sphingosine-1-phosphate receptor-modulation. Although fingolimod-associated lymphopenia is a well-known phenomenon, the exact cause for the intra- and inter-individual differences of the fluctuation of lymphocyte count and its subtypes is still subject of debate. In this analysis, we aim to estimate the significance of the individual variation of distinct lymphocyte subsets for differences in absolute lymphocyte decrease in fingolimod treated patients and discuss how different lymphocyte subset patterns are related to clinical presentation in a long-term real life setting.

**Methods/Design:**

One hundred and thirteen patients with MS were characterized by complete blood cell count and immune cell phentopying of peripheral lymphocyte subsets before, at month 1 and every 3 months up to 36 months of fingolimod treatment. In addition, patients were monitored regarding clinical parameters (relapses, disability, MRI).

**Results:**

There was no significant association of baseline lymphocyte count and lymphocyte subtypes with lymphocyte decrease after fingolimod start. The initial drop of the absolute lymphocyte count could not predict the level of lymphocyte count during steady state on fingolimod. Variable CD8+ T cell and NK cell counts account for the remarkable intra- and inter-individual differences regarding initial drop and steady state level of lymphocyte count during fingolimod treatment, whereas CD4+ T cells and B cells mostly present a quite uniform decrease in all treated patients. Selected patients with lymphocyte count >1.0 GPT/l differed by higher CD8+ T cells and NK cell counts compared to lymphopenic patients but presented comparable clinical effectiveness during treatment.

**Conclusion:**

Monitoring of the absolute lymphocyte count at steady state seems to be a rough estimate of fingolimod induced lymphocyte redistribution. Our results suggest, that evaluation of distinct lymphocyte subsets as CD4+ T cells allow a more detailed evaluation to weigh and interpret degree of lymphopenia and treatment response in fingolimod treated patients.

## Introduction

Multiple sclerosis (MS) is a chronic inflammatory disease of the central nervous system (CNS) initiated and perpetuated by an imbalance in the immune-regulatory network. Different MS specific treatment regimens are available and aim to govern autoimmunity and CNS inflammation (1). For some years cellular anti-migratory strategies have been used to control cell migration and accumulation in the CNS (2). Fingolimod acts as sphingosine-1-phosphate (S1P) receptor modulator that inhibits S1P-mediated lymphocyte egress from lymph nodes impairing peripheral lymphocyte recirculation (3–5). This unique mechanism of action results in reduction of absolute lymphocyte counts including specific subsets as naïve T cells, central memory T and B cells but also pro-inflammatory Th1 and Th17 cell subsets in the peripheral and central compartment (3, 6–9). Natalizumab is a further effective therapy for MS patients also known by its anti-migratory mechanism of action. Compared to fingolimod, natalizumab is effective by the block of the α4-subunit of the very late antigen-4 that impairs transmigration of immune cells across the blood-brain barrier into the CNS. Although immune cell subsets are rapidly decreased in the CNS compartment as well, natalizumab lead to significant lymphocyte increase and distinct changes in CD4/CD8 ratio in peripheral blood of treated patients (10, 11). In contrast to other MS treatment regimens e.g., dimethylfumarate therapy even low levels of absolute lymphocyte count up to 0.2 GPT/l can be tolerated during fingolimod treatment (12–16). Other disease managing regimens e.g., in cancer treatment use scoring systems as the National Cancer Institute Common Terminology Criteria for Adverse Events (NCI-CTAE) for grading degree and severity of adverse events including lymphopenia to define risk of infectious complications (17). Interestingly, even though there is a potential increased risk for severe and opportunistic infections due to persistent decrease of CD4+ T cells, only herpes reactivation and infection is relevantly increased in fingolimod treatment (18). Although the peripheral decreased lymphocyte count is a well-known phenomenon for clinicians who are experienced with fingolimod, the exact details of the intra- and inter-individual differences regarding drop and fluctuation of lymphocyte count as well of its subtypes is still a subject of debate (19–22). Up to now, it is not clear which factors can lead to higher vs. lower lymphocyte counts during fingolimod treatment and whether distinct lymphocyte count patterns can assist to select patients that are at higher risk for infections or non-responsiveness to fingolimod treatment (20, 21, 23).

Real-world evidence (RWE) and observational studies are becoming increasingly popular because they provide longitudinal information on usefulness of drugs in real life and have the ability to discover uncommon or rare adverse drug reactions inclusive lab abnormalities (24, 25). Following this approach of real world lab data, here we aim to estimate the significance of the individual variation of distinct lymphocyte subsets for differences in absolute lymphocyte count decrease in fingolimod treated patients and discuss how different lymphocyte subset patterns are related to clinical presentation.

## Methods

### Patients

In our observational real world cohort, we included 113 RRMS patients that were treated in our MS center in Dresden (65 females/48 males) with highly active disease course (Supplementary Table 1). After critical review of all relevant clinical and imaging parameters and available treatment options, fingolimod treatment was initiated at a dose of 0.5 mg fingolimod daily. Before fingolimod start, 80.5% of patients were pre-treated with different DMTs (Supplementary Table 1). During a standardized treatment switch procedure, injectables were stopped 2 weeks before fingolimod start, natalizumab was stopped 12 weeks before fingolimod start and other DMTs at least 6 months before fingolimod start. Blood samples were collected before (baseline), at month 1 and every 3 months up to 36 months of fingolimod treatment. Additionally patients were monitored regarding clinical parameters including infections, relapse activity, confirmed disability progression measured by EDSS (≥1.0 point increase if EDSS baseline score was < 4.0; ≥0.5 point increase if EDSS baseline score was ≥4.0) and BMI three-monthly and MRI progression every year assessed by an examined neuro-radiologist. MRI progression was defined in case of appearance of new gadolinium enhancing lesions or new T2 lesions in cerebral MRI scan. No serious adverse events appeared in our cohort. Data have been collected from the MSDS3D database. The study was approved by the institutional review board of the University Hospital of Dresden. Patients gave their written informed consent.

### Routine blood analysis

Standardized blood testing was performed for routine blood parameters at the Institute of Clinical Chemistry and Laboratory Medicine, University Hospital in Dresden, Germany. The institute complies with standards required by DIN-EN-ISO-15189:2014 for medical laboratories. Whole blood samples were collected in ethylene diamine tetra acetic acid (EDTA). Routine blood testing included complete blood cell count.

### Immune cell phenotyping by fluorescence-activated cell sorting (FACS)

Whole blood samples were collected in EDTA. After collection blood samples were incubated with fluorescence labeled monoclonal antibodies including anti-CD3, anti-CD4, anti-CD8, anti-CD16, anti-CD19, anti-CD56 (BD Biosciences, Heidelberg, Germany) to define T cell, B cell, and natural killer (NK) cell subpopulations. Afterwards, red blood cells were lysed using BD FACS Lysing Solution (BD Bioscience). After washing with FACS buffer (phosphate buffered saline, 0.2% fetal bovine serum, 0.02% sodium azide, all Biochrom) cells were evaluated on FACSCanto II flow cytometer.

### Evaluations and definition of the groups

Different approaches were chosen to discuss our study objectives: (1) the whole cohort was examined to evaluate mean changes in peripheral immune cell subsets on group level. Ten patients out of our cohort presented lymphocyte count >1.0 GPT/L after fingolimod start. In addition, matched groups of patients with lymphocyte count 0.5–1.0 GPT/l and ≤ 0.5 GPT/l were defined and used for further considerations. Additionally intra-individual variability of lymphocytes and its subsets was evaluated in these subgroups. Intra-individual variability was calculated as the standard deviation of absolute cell counts measured every 3 months between month 1 to month 36 of fingolimod therapy in each patient (2). The impact of initial absolute lymphocyte drop was assessed. Median-split was done for the initial absolute drop of lymphocyte count at 1.40 GPT/l and the whole cohort was divided into a high vs. low lymphocyte drop group (3). For the third approach the relevance of high vs. low steady state lymphopenia during fingolimod treatment was assessed. Median-split for lymphocyte count was calculated for the steady state period after month 6 at 0.48GPT/l and a lower steady state group vs. a higher steady state group was selected out of the whole cohort.

Scoring and grading of the level of lymphopenia was performed using the system of the National Cancer Institute Common Terminology Criteria for Adverse Events (NCI-CTAE). Characterization of levels of lymphopenia using NCI-CTAE grading was defined by lymphopenia grade 1: > 0.8 GPt/L, lymphopenia grade 2: 0.5-0.8 GPt/L, lymphopenia grade 3: 0.2-0.5 GPt/L, and lymphopenia grade 4: < 0.2 GPt/L.

### Statistical analysis

Data were analyzed applying Generalized Linear Mixed Models (GLMM) with Gamma distribution and log link function for evaluations with immune cell populations with skewed distribution. Absolute lymphocyte counts and lymphocyte steady state were skewed distributed and though groups of interest were defined using medians-split to define groups of same and comparable sample size. Although lymphocyte count drop was normal distributed medians-split was applied as well to use comparable methodology. Differences in patient characteristics were defined using Analysis of Variances (ANOVA), Kruskal-Wallis H test, Person's chi-square test or Fischer exact test, and an alpha error level of 5%. Correlation was calculated using Spearman's correlation. In order to control for the familywise error rate of the multiple GLMM analyses, comparable alpha error rates were adjusted via Bonferroni correction as α/k were k is the number of analyses per parameter. Therefore, values of *p* < 0.0125 (0.05/4) were considered significant.

## Results

### Lymphocyte decrease and its relevance in lymphocyte variation during fingolimod treatment

**Figure 1:**
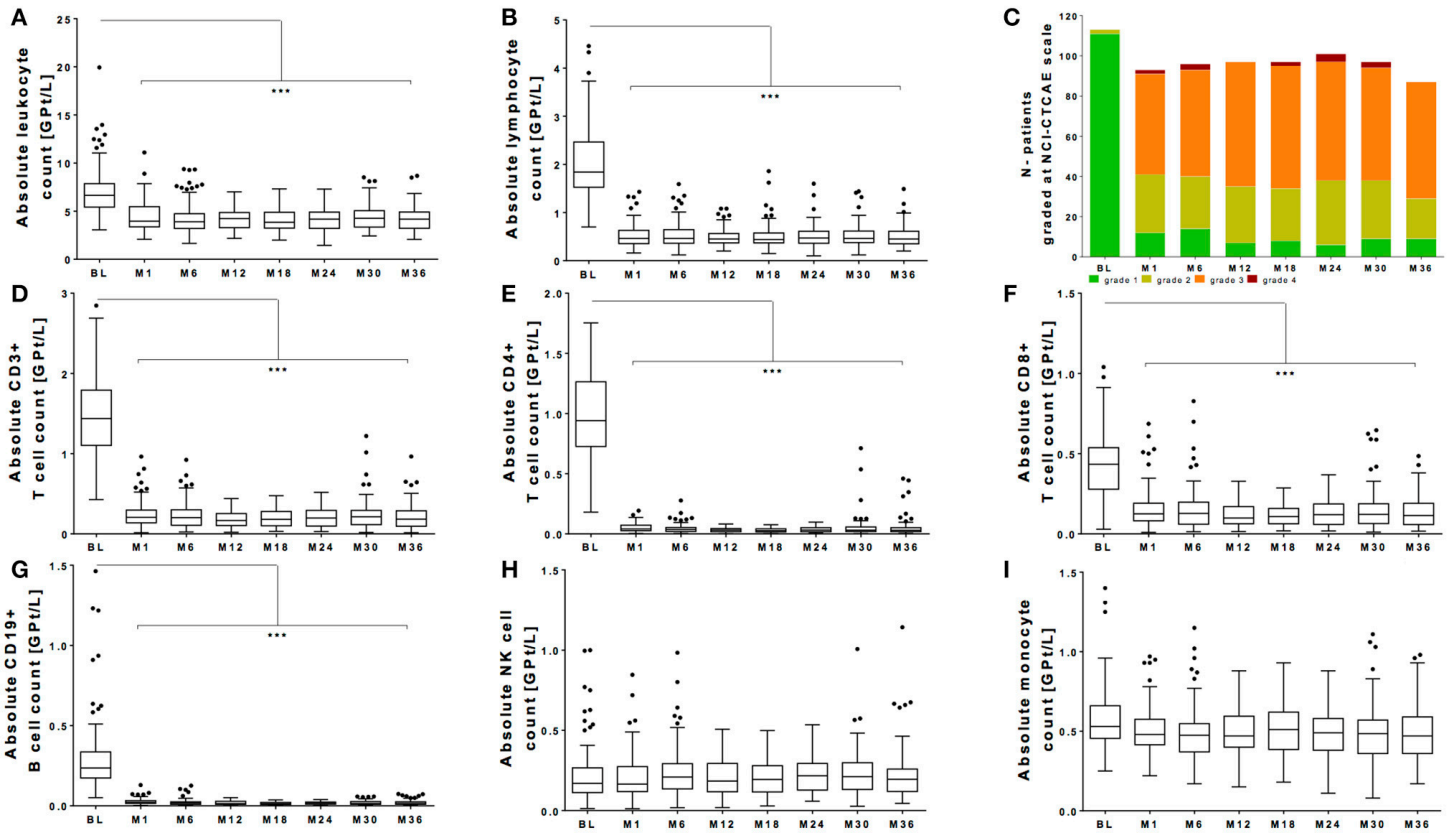
Absolute count of peripheral white blood cells during fingolimod treatment. Absolute cell counts of leukocytes **(A)** lymphocytes **(B)**, CD3+ T cells **(D)**, CD4+ T cells **(E)**, CD8+ T cells **(F)**, CD19+ B cells **(G)** and NK cells **(H)**, and monocytes **(I)** are depicted. Data for the whole cohort are shown as Boxplot Tukey before fingolimod start (baseline, BL), month 1 and every 6 months follow up. **(C)** Distribution of different ranges of lymphocyte count are shown graded with NCI-CTCAE: lymphopenia grade 1 >0.8 GPt/L (green), lymphopenia grade 2 0.5-0.8 GPt/L (yellow), lymphopenia grade 3 0.2-0.5 GPt/L (orange) and lymphopenia grade 4 < 0.2 GPt/L (red). Asterisks indicate level of significance of pairwise comparison (^***^p < 0.001).

All patients of our observational cohort demonstrated the well-known drop of absolute lymphocyte count after fingolimod initiation. There was a significant drop of leukocyte count and lymphocyte count (Figures 1A,B). Evaluating grading by NCI-CTAE demonstrated that most of the patients presented lymphopenia grade 2 or 3 after fingolimod start (Figure 1C). NCI-CTAE grade 4 was reached only at single time points in selected patients. None of the patients stopped fingolimod treatment due to lymphopenia during the observation period as retest revealed grade 3 lymphopenia. Monocytes and NK cells changed only mildly (Figures 1H,I), whereas the most intense decrease was seen on T and B cell subtypes (Figures 1D–G).

**Figure 2:**
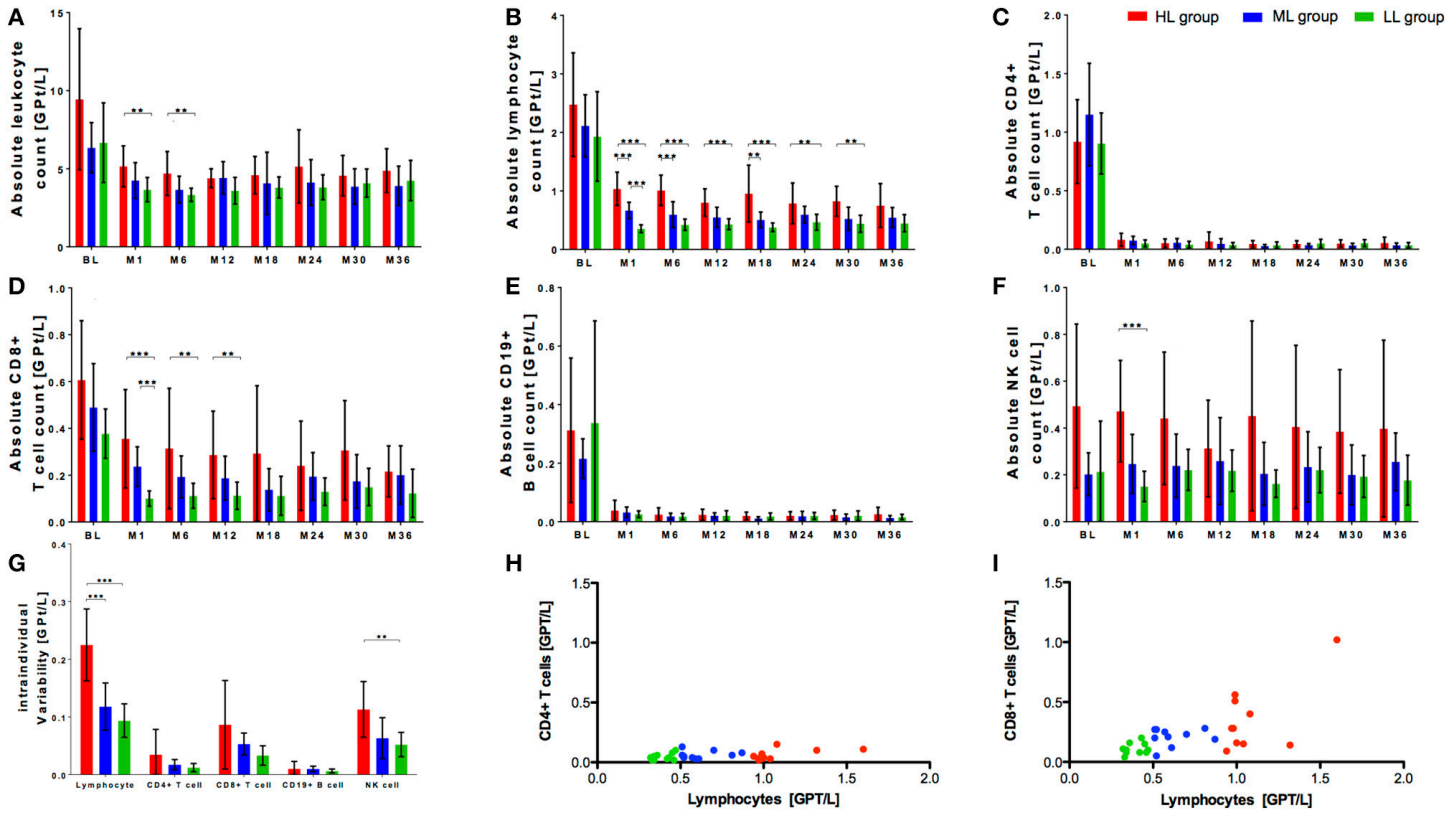
Cell count of selective immune cell subpopulations in cohorts with different initial lymphocyte decrease after fingolimod start. Absolute cell count of leukocytes **(A)**, lymphocytes **(B)**, CD4+ T cells **(C)**, CD8+ T cells **(D)**, CD19+ B cells **(E)**, and NK cells **(F)** are depicted. Three groups of patients during fingolimod treatments are compared: HL group (high lymphocyte count 1 ≥ 1.0 GPt/L, red) ML group (medium lymphocyte count 0.5–1.0 GPt/L, blue) and LL group (low lymphocyte count ≤ 0.5 GPt/L, green). BL, month 1 and 6 months interval of evaluation up to 36 months of fingolimod therapy are depicted. **(G)** Intra-individual variability of lymphocytes, CD4+ T cells and CD8+ T cells, CD19+ B cells and NK cells compared between HL group, ML group and LL group are presented. Intra-individual variability was declared as the standard deviation of absolute cell counts between month 1 to month 36 of fingolimod therapy. Mean ± SD are depicted. Asterisks indicate level of significance of pairwise comparison. (^**^p < 0.01 and ^***^p < 0.001) **(H,I)** Correlation of decrease of CD4+ T cells resp. CD8+ T cells and lymphocytes are presented. Mean of absolute lymphocyte count, CD4+ T cell count and CD8+ T cell count between month 1, month 3, and month 6 after fingolimod start were calculated and depicted. Level of statistical significance was evaluated using Spearman's correlation. **(H)** *r* = 0.2849, n.s. **(I)** *r* = 0.5998, *p* < 0.001.

Within our cohort, 10 of 113 patients presented with lymphocyte counts ≥1.0 GPT/L. This specific high lymphocyte group (HL) was compared with a matched (sex, age) fingolimod treated patient group with lymphocyte counts of 0.5-1.0 GPT/l (median lymphocyte group, ML) respective ≤ 0.5 GPT/l (low lymphocyte group, LL) (Table 1). Although characterized by varying levels in lymphocyte decrease, the patients did not differ regard clinical parameters including relapse activity, confirmed EDSS progression and MRI progression or occurrence of reported infectious events between all three groups (Table 1). Distribution of previous DMT use was different in all three groups with a higher proportion of interferon-beta use (30–40%) in the ML and LL group whereas glatiramer acetate was used more frequent in the HL group before fingolimod start (Table 1). At baseline, there was a trend to a higher absolute count of leukocytes and lymphocytes in HL group compared to ML and LL group. Nevertheless, this trend was not statistical significant (Figures 2A,B). After fingolimod start, all lymphocyte counts significantly decreased (Table 2). The HL group presented with the highest lymphocyte count at month 1. Thereafter, lymphocyte count decreased further on but was still higher and different compared to lymphocyte counts of ML and LL group (Figure 2B, Table 2). Additionally, intra-individual variability was evaluated in all three groups: there was a wide intra-individual variation in lymphocyte count in HL group after month 1 (Figure 2G). After the initial drop, ML group and LL group presented with quiet stable levels of lymphocyte count over the whole observation period (Figure 2B). Intra-individual variation of lymphocyte count presented at a smaller range compared to HL group (Figure 2G).

**Table 1:** Patient characteristics.

	**HL**	**ML**	**LL**
Age (yr ± SD)	36.6 (9.9)	39.2 (8.6)	39.0 (8.1)
Duration since MS diagnosis (yr ± SD)	4.8 (2.1)	3.6 (4.0)	5.0 (3.6)
Sex (f/m)	3/7	3/7	3/7
Previous DMT use before fingolimod start [no. (%)]	10 (100)	7 (70)	7 (70)
Interferon beta	–	4 (40)	3 (26)
Glatirameracetat	8 (80)	3 (26)	3 (26)
Natalizumab	1 (10)	–	1 (10)
Others	1 (10)	–	–
None previous DMT use [no. (%)]	–	3 (26)	3 (26)
BMI at BL (± SD)	23.8 (2.5)	27.1 (4.0)	25.9 (4.8)
EDSS at BL (± SD)	2.4 (1.0)	2.0 (0.6)	3.6 (1.6)
Relapses during fingolimod (no.)	0	0	0
Confirmed EDSS progression during fingolimod (no.)	0	0	1
MRI progression during fingolimod (no.)	4	2	3
Adverse events—infections (no.)	23	14	15

*Baseline characteristics of evaluated patients are depicted. HL (high lymphocyte group) includes patients with lymphocyte count ≥ 1.0 GPT/L after fingolimod start. ML (median lymphocyte group) with lymphocyte count of 0.5–1.0 GPT/l and LL (low lymphocyte group) with lymphocyte count ≤ 0.5 GPT/l. DMT, disease modifying treatment. Other previous treatments included: Laquinimod. Number of relapses, confirmed EDSS progression, MRI progression and infectious events during fingolimod treatment period are presented. Yr, year; SD, standard deviation; f, female; m, male; BL, baseline; no, number*.

**Table 2:** Level of significance for comparing the groups—global effects.

**HL/ML/LL group**	**Group-effect**	**Time-effect**	**Time-by-group interaction**	**HAD/LAD group**	**Group-effect**	**Time-effect**	**Time-by-group interaction**	**HSS/LSS group**	**Group-effect**	**Time-effect**	**Time-by-group interaction**
Leukocyte	*p* < 0.001	*p* < 0.001	*p* = 0.747	Leukocyte	*p* < 0.001	*p* < 0.001	*p* = 0.566	Leukocyte	*p* < 0.001	*p* < 0.001	*p* = 0.845
Lymphocyte	*p* < 0.001	*p* < 0.001	*p* = 0.008	Lymphocyte	*p* = 0.120	*p* < 0.001	*p* < 0.001	Lymphocyte	*p* < 0.001	*p* < 0.001	*p* < 0.001
CD3+ T cell	*p* < 0.001	*p* < 0.001	*p* = 0.040	CD3+ T cell	*p* = 0.236	*p* < 0.001	*p* < 0.001	CD3+ T cell	*p* < 0.001	*p* < 0.001	*p* < 0.001
CD4+ T cell	*p* = 0.048	*p* < 0.001	*p* = 0.264	CD4+ T cell	*p* = 0.381	*p* < 0.001	*p* < 0.001	CD4+ T cell	*p* < 0.001	*p* < 0.001	*p* = 0.013
CD8+ T cell	*p* < 0.001	*p* < 0.001	*p* = 0.450	CD8+ T cell	*p* = 0.383	*p* < 0.001	*p* < 0.001	CD8+ T cell	*p* < 0.001	*p* < 0.001	*p* = 0.073
CD19+ B cell	*p* = 0.017	*p* < 0.001	*p* = 0.957	CD19+ B cell	*p* < 0.001	*p* < 0.001	*p* = 0.004	CD19+ B cell	*p* = 0.043	*p* < 0.001	*p* = 0.877
Monocyte	*p* < 0.001	*p* = 0.558	*p* = 0.926	Monocyte	*p* = 0.002	*p* = 0.005	*p* = 0.592	Monocyte	*p* < 0.001	*p* = 0.003	*p* = 0.869
NK cell	*p* < 0.001	*p* = 0.981	*p* = 0.855	NK cell	*p* = 0.220	*p* = 0.955	*p* = 0.165	NK cell	*p* < 0.001	*p* = 0.811	*p* = 0.995

*Values of p < 0.0125 were considered significant*.

**Figure 3:**
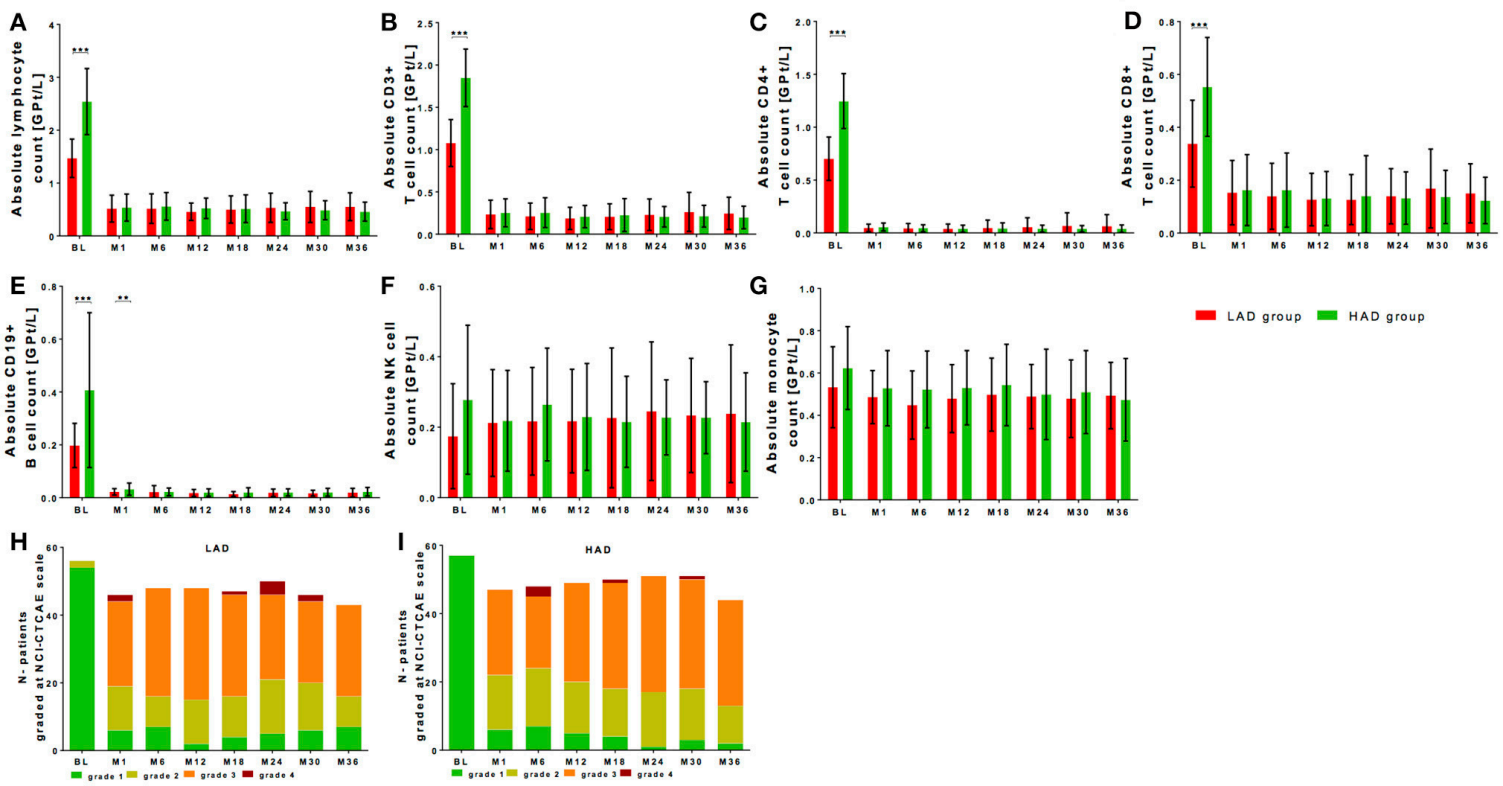
Cell count of selective immune cell subpopulations in patients with high (HAD) vs. low absolute lymphocyte (HAD) drop. Absolute cell counts of lymphocytes **(A)**, CD3+ T cells **(B)**, CD4+ T cells **(C)**, CD8+ T cells **(D)**, CD19+ B cells **(E)**, NK-cells **(F)** and monocytes **(G)** are depicted. Comparison between two groups is presented: LAD group is defined as lower absolute lymphocyte drop group (< 1.40 GPT/l, red) and HAD group defined as higher lymphocyte drop group (≥ 1.40 GPT/l, green) after median-split. Data are shown before fingolimod start (baseline, BL), month 1 and every 6 months follow up. Mean ± SD are depicted. **(H,I)** Distribution of different ranges of lymphocyte count are shown graded with NCI-CTCAE: lymphopenia grade 1 >0.8 GPt/L (green), lymphopenia grade 2 0.5-0.8 GPt/L (yellow), lymphopenia grade 3 0.2-0.5 GPt/L (orange) and lymphopenia grade 4 < 0.2 GPt/L (red). Results are presented for LAD group **(H)** and HAD group **(I)** threw an observation period of 36 months. Asterisks indicate level of significance of pairwise comparison (^**^p < 0.01 and ^***^p < 0.001).

Lymphocyte subpopulations have been analyzed in all three groups: there were no significant differences at baseline between the three groups for CD4+ T cells and CD19+ B cells (Figures 3C,E). In contrast, highest CD8+ T cell and NK cell count was seen in HL group before fingolimod start (Figures 2D,F). After fingolimod start, T and B cell subsets significantly dropped in all groups (Figures 2C–E, Table 2). Interestingly, CD4+ T cells and CD19 B cells dropped similarly and did not differ even at steady state in all three groups (Figures 2C,E, Table 2), while CD8+ T cells were constantly higher in HL group compared to ML and LL group during fingolimod treatment (Figure 2D, Table 2). NK cells were not affected by fingolimod treatment but significantly different in absolute count between the HL, ML, and LL group at baseline and follow up (Figure 2F, Table 2). Evaluation of intra-individual variability of lymphocyte subtypes demonstrated high variability in CD8+ T cells and NK cells especially in HL group whereas ML and LL group presented comparable and lower intra-individual variability (Figure 2G). CD4+ T cells and CD19+ B cells were characterized by low variability reflecting constant and stable levels within observation period in all investigated groups (Figure 2G). Additional correlation analyses demonstrated that there was a comparable CD4+ T cell decrease irrespective of the lymphocyte count (*r* = 0.2849, n.s.; Figure 2H) whereas CD8+ T cell decrease was strongly correlated with lymphocyte decrease in our patients (*r* = 0.5998, *p* < 0.001; Figure 2I). These data indicate that variability and occasionally high levels of absolute lymphocyte count are primarily caused by the wide individual variation in CD8+ T cell count, while CD4+ T cells are more robust and less variable to evaluate fingolimod treatment effects.

### Initial drop of lymphocyte count does not predict long-term level of lymphocyte count

Lymphocyte decrease dependent on individual lymphocyte drop during fingolimod treatment is frequently discussed. Initial drop of lymphocyte count was individually calculated using lymphocyte count at baseline vs. steady state after 6 months for each patient. Median-split was performed for the initial absolute drop of lymphocyte count resulting at 1.40 GPT/l. A lower absolute drop group (LAD group; < 1.40 GPT/l) and a higher absolute drop group (HAD group; ≥ 1.40 GPT/l) were defined. Both groups were not significantly different in age, sex, disease duration, BMI, or EDSS at baseline (Table 2). In the HAD there was a significant higher number of patients that were pretreated with natalizumab (21.1% in the HAD vs. 3.6% in the LAD group, *p* < 0.01).

At baseline, absolute lymphocyte count was significantly higher in the HAD group (Figure 3A, Table 2). Highest baseline lymphocyte counts were seen in previously natalizumab treated patients (treatment stopped 12 weeks before fingolimod initiation), whereas lymphocyte counts in patients with other DMT use or none previous treatments were at comparable range. By definition, the HAD group demonstrated significant higher absolute as well as relative lymphocyte drop compared to LAD group (Table 3, *p* < 0.001). Highest drop was found in patients with previous natalizumab treatment (mean absolute drop 2.52 GPT/l ± SD 1.00 GPT/l). During long-term observation, lymphocyte counts were significantly reduced to comparable levels in the LAD group vs. HAD group (Figure 3A, Table 3). There was no significant correlation between absolute lymphocyte drop and absolute lymphocyte count at steady state (*r* = −0.041, *p* = 0.668). Additional characterization of levels of lymphopenia using NCI-CTAE grading demonstrated that there was a similar distribution of grades of lymphopenia between the LAD group and HAD group threw the whole observation period (Figures 3H,I). There was no association between type of previous DMT use and grade of lymphopenia during fingolimod therapy. Independent of degree of lymphocyte drop, disease activity parameters including relapse activity, disability progression and MRI progression were comparable in both groups (Table 3). Furthermore, no significant differences in BMI or occurrence of acute infections in the LAD vs. HAD group could be confirmed.

**Table 3:** Patient characteristics.

	**LAD group**	**HAD group**
*N* patients	56	57
Age (yr ± SD)	39.9 (9.6)	39.0 (10.1)
Duration since MS diagnosis (yr ± SD)	6.4 (5.6)	7.1 (6.1)
Sex (f/m)	28/28	37/20
Previous DMT use before fingolimod start [no. (%)]	43 (76.8)	48 (84.2)
Interferon beta	23 (41.1)	22 (38.6)
Glatirameracetat	14 (25.0)	13 (22.8)
Natalizumab	2 (3.6)	12 (21.1)
Others	4 (7.1)	1 (1.8)
None previous DMT use [no. (%)]	13 (23.2)	9 (15.8)
BMI—BL (± SD)	24.1 (4.0)	25.6 (5.1)
EDSS—BL (± SD)	3.0 (1.6)	2.7 (1.3)
Lymphocyte absolute drop [GPT/L] Mean (95% CI)	0.95 (0.87; 1.03)	2.04 (1.88; 2.20)
Lymphocyte relative drop [%] Mean (95% CI)	64.01 (60.80; 67.21)	79.65 (78.03; 81.26)
Lymphocyte steady state [GPT/L] Mean (95% CI)	0.52 (0.46; 0.57)	0.50 (0.46; 0.55)
Relapse during fingolimod (no.)	13	12
Confirmed EDSS progression during fingolimod (no.)	9	11
MR progression during fingolimod (no.)	18	17
Adverse events—infections (no.)	38	40

*Patient characteristics of evaluated patients are depicted. LAD group (lower absolute drop group) includes patients with an absolute lymphocyte drop < 1.40 GPT/l. HAD includes patients with an absolute lymphocyte drop ≥ 1.40 GPT/l. DMT, disease modifying treatment. Other previous treatments included: Azathioprin (2), Laquinimod (3). Number of relapses, confirmed EDSS progression, MRI progression and infectious events during fingolimod treatment period are presented. Yr, year; SD, standard deviation; f, female; m, male; BL, baseline; no, number*.

Evaluation of lymphocyte subtypes demonstrated that T and B cells presented with significantly higher absolute cell counts at baseline in the HAD group (Figures 3B–E, Table 3). After significant drop, T and B cells were at comparable levels in both groups during fingolimod treatment (Figures 3B–E, Table 3). There were no differences in absolute NK cell at baseline and after treatment initiation between HAD and LAD group (Figure 3F, Table 3). In monocytes only mild changes were found during fingolimod treatment in both groups (Figure 3G, Table 3).

### Lymphocyte subtypes differ in patients with high-level vs. low-level steady state lymphopenia

It is known that lymphocyte count and changes of its subpopulations reach at latest their steady state 6 months after fingolimod start. Median-split was calculated for the steady state period after month 6 (lymphocyte count of 0.48GPT/l). A lower steady state group (LSS group; < 0.48 GPT/l) and a higher steady state group (HSS group; ≥ 0.48 GPT/l) was defined. There were no significant differences in age, disease duration, previous DMT use, EDSS or BMI at baseline (Table 3). In the LSS group a significant higher number of women (LSS group 70.3% vs. HSS group 45.7%) could be identified (*p* < 0.05).

**Figure 4:**
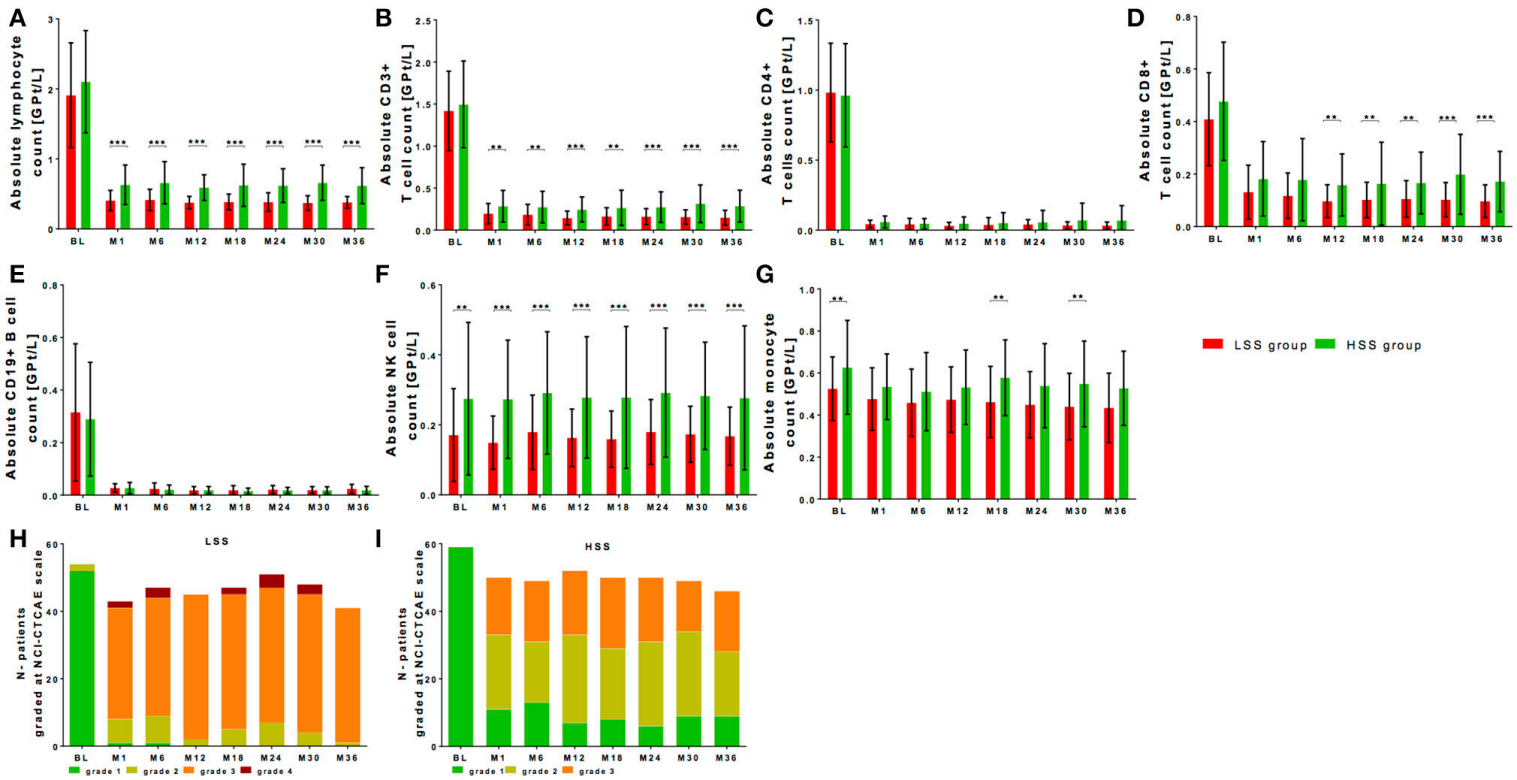
Cell count of selective immune cell subpopulations in patients with high-level vs. low-level steady-state lymphopenia. Absolute cell counts of lymphocytes **(A)**, CD3+ T cells **(B)**, CD4+ T cells **(C)**, CD8+ T cells **(D)**, CD19+ B cells **(E)**, NK cells **(F)**, and monocytes **(G)** are depicted. Comparison between two groups is presented: LSS group defined as lower steady state group (< 0.48 GPT/l; red) and HSS group defined as higher steady state group (≥ 0.48 GPT/l; green) after median split. Data are shown before fingolimod start (baseline, BL), month 1 and every 6 month follow up. Mean ± SD are depicted. **(H,I)** Distribution of different ranges of lymphocyte count are shown graded with NCI-CTCAE: lymphopenia grade 1 >0,8 GPt/L (green), lymphopenia grade 2 0,5-0,8 GPt/L (yellow), lymphopenia grade 3 0,2-0,5 GPt/L (orange) and lymphopenia grade 4 < 0,2 GPt/L (red). Results are presented for LSS group **(H)** and HSS group **(I)** threw an observation period of 36 months. Asterisks indicate level of significance of pairwise comparison (^**^p < 0.01 and ^***^p < 0.001).

Baseline lymphocyte levels demonstrated only a trend to higher baseline lymphocyte counts in the HSS group that were significantly decreased to different levels of lymphocyte steady state in both groups (Figure 4A, Table 2). As well after fingolimod start, the absolute lymphocyte drop was not different between LSS group and HSS group in reference to baseline levels; a higher significant relative lymphocyte drop (*p* < 0.001) could be shown for LSS group (Table 4). Grading with NCI-CTAE scale demonstrated that LSS group included single patients that presented with lymphocyte counts at grade 2 already before fingolimod start (Figure 4H). All of these patients were pretreated with interferon-beta treatment that was stopped two weeks before fingolimod start. During follow up, almost all patients in the LSS group presented with lymphocyte counts lower than grade 2 and up to grade 4 (Figure 4H). None of these patients stopped fingolimod treatment due to lymphopenia during the observation period as retest revealed grade 3 lymphopenia. HSS group included only patients with lymphocyte levels in the reference range before fingolimod start and none of the patients presented lymphocyte levels lower than grade 3 during follow up (Figure 4I). There was no association between type of previous DMT use and grade of lymphopenia during fingolimod therapy. Patients that presented lymphocyte counts at lower NCI-CTAE grade were not at risk for increased occurrence of infections compared to patients with higher lymphocyte count grade (Table 4). We could not find any differences in BMI between the LSS group and HSS group. There were no significant differences in clinical progression or MRI activity over the 36 months observation period in LSS vs. HSS patients (Table 4).

**Table 4:** Patient characteristics.

	**LSS group**	**HSS group**
*N* patients	54	59
Age (yr ± SD)	40.4 (10.0)	38.6 (9.6)
Duration since MS diagnosis (yr ± SD)	7.7 (6.0)	5.7 (5.6)
Sex (f/m)	38/16	27/32
Previous DMT use before fingolimod start [no. (%)]	45 (83.3)	46 (78.0)
Interferon beta	25 (46.3)	20 (33.9)
Glatirameracetat	10 (18.5)	17 (28.8)
Natalizumab	6 (11.1)	8 (13.6)
Others	4 (7.4)	1 (1.7)
None previous DMT use [no. (%)]	9 (16.7)	13 (22.0)
BMI—Baseline (± SD)	24.5 (4.6)	25.3 (4.7)
EDSS—Baseline (± SD)	3.0 (1.5)	2.7 (1.3)
Lymphocyte absolute drop [GPT/L] Mean (95% CI)	1.52 (1.32;1.73)	1.48 (1.29;1.66)
Lymphocyte relative drop [%] Mean (95% CI)	76.86 (74.05;79.68)	67.35 (64.16;70.54)
Lymphocyte steady state [GPT/L] Mean (95% CI)	0.38 (0.37;0.40)	0.63 (0.58;0.68)
Relapse during fingolimod (no.)	15	10
Confirmed EDSS progression during fingolimod (no.)	13	7
MR progression during fingolimod (no.)	15	20
Adverse events—infections (no.)	39	39

*Patient characteristics of evaluated patients are depicted. LSS group (lower steady state group) includes patients with a lymphocyte count at steady state < 0.48 GPT/l. HSS group (higher steady state group) includes patients with a lymphocyte count at steady state ≥ 0.48 GPT/l. Number of relapses, confirmed EDSS progression, MRI progression and infectious events during fingolimod treatment period are presented. Yr, year; SD, standard deviation; f, female; m, male; BL, baseline; no, number*.

There were no significant differences regarding baseline levels of all analyzed T and B cell subpopulations between LSS group and HSS group (Figures 4B–E). After fingolimod start, CD4+ T cells and CD19+ B cells significantly decreased to comparable levels, whereas CD8+ T cells were significantly higher in the HSS group vs. LSS group (Figures 4C–E, Table 2). In addition, NK cells presented with higher counts at baseline and during treatment period in HSS patients (Figure 4F, Table 2). Interestingly, monocytes were higher at baseline and follow up in the HSS compared to the LSS group (Figure 4G, Table 2).

## Discussion

Lymphopenia is an integral part of fingolimod therapy based on its unique mechanism of action (8). Already initial clinical trials reported a decrease of lymphocyte count about 70% and discussed wide inter-individual differences in lymphocyte drop and fluctuation of total lymphocyte count in treated patients (27–29) There are different hypotheses which try to interpret the variation in levels of lymphopenia in fingolimod treated patients (19, 23, 30). Previous reports already discussed no relevant relation between degree of lymphopenia and clinical efficacy as well as occurrence of side effects (21, 28). Up to date it is unclear whether and why patients appear with less or marked decrease in lymphocyte count after fingolimod start.

Real-world data provide longitudinal information on different outcomes including effects on lab parameters (24). Implementing lab data into a comprehensive real world data approach can complete the fundamental quest of real world evidence for individually improved treatment decisions and balanced therapeutic risk assessment. In our observation, we evaluated individual variation of lymphocytes and its subsets in a long-term real life setting. There was a wide range of baseline lymphocyte levels in our cohort. Differences in baseline lymphocyte count dependent on previous DMT use cannot be excluded based on restricted washout periods. In our evaluation, moderate lymphopenia at baseline was more frequent in interferon-beta treated patients whereas natalizumab pretreatment lead to higher levels at fingolimod start. Other reports discussed that higher baseline lymphocyte levels were associated with higher lymphocyte count threw follow up (20, 22). Furthermore, increased risk of fingolinod-associated lymphopenia in patients with interferon-beta pretreatment was suggested (20). In our cohort, we could not statistically prove that higher baseline levels or different pretreatment conditions were associated with the level of lymphocyte count or decrease at steady state during fingolimod therapy. Previous reports discussed an increased risk of lymphopenia in patients with BMI < 18.5 kg/m^2^ (20). Others presented data that could not confirm correlation between BMI and lymphopenia levels (22). In our observation, we could not define a significant relation between BMI and lymphocyte count, probably because only four patients presented persistent BMI levels < 18.5 kg/m^2^. Nevertheless, its lymphocyte count was reduced about 0.39–1.13 GPT/l at comparable range in contrast to other treated patients.

Interestingly, the absolute and relative lymphocyte drop after fingolimod start could not predict the level of lymphopenia in our cohort. Although marked differences in lymphocyte drop were present between patients, lymphocyte levels reached comparable levels independent of degree of lymphocyte drop. Most of the recent reports evaluating lymphopenia and its variation in fingolimod treated patients did not analyze specific lymphocyte subtypes. Although absolute lymphocyte count differed in our patients, additional analysis of lymphocyte subtypes confirmed that CD4+ T cells and CD19+ B cells were decreased at a comparable level with narrow intra- and inter-individual variation in all fingolimod treated patients irrespective of the degree of lymphopenia.

In line with previous data, absolute NK cells count was not altered by fingolimod treatment in our cohort (26). NK cell recirculation is mediated by S1PR1 and S1PR5. It is suggested that S1PR5 is less susceptible to fingolimod than S1PR1, which is used by the other lymphocyte subtypes (31). These circulating NK cells maintain their functional capacity and contribute to the immunosurveillance by the innate immune system in fingolimod treated patients (32). In our study, patients with lower lymphocyte count during fingolimod treatment were associated with significant lower NK cell number. Intra-individual fluctuation of NK cell count was highest in patients with higher level of lymphocyte count compared to patients with lower level of lymphocyte count and did significantly differ compared to CD4+ T cells and CD19+ B cells. Main differences in absolute lymphocyte count were induced by variation in NK cell count.

Although initially supposed, degree of lymphopenia could not confirm a correlation with clinical treatment response (21). Others discussed increased CD3+ and CD8+ T cell counts and decreased number of NK cells in first 6 months of fingolimod therapy as predictive marker for relapse activity (23). In our cohort, some patients presented with lymphocyte counts above 1.0 GPT/l. In these patients we could not confirm differences of clinical MS disease activity compared to patients with < 1.0 GPT/l or even < 0.5 GPT/l lymphopenia. Differences in lymphocyte count were caused by higher levels and of CD8+ T cell and NK cell count in higher lymphocyte cohort whereas CD4+ T cells and CD19+ B cells were markedly and comparable decreased in all three groups. Though, we suppose that decrease in CD4+ T cell count is associated with fingolimod efficacy and response rather than complete lymphocyte count.

During fingolimod treatment, the degree of lymphopenia and its clinical relevance defined by increased risk of infectious events is critically discussed (18, 30). Grading the level of lymphopenia using the NCI-CTAE is done especially for oncological diseases and treatment to define patients at higher risk for infections (17). Level of lymphopenia displayed in our cohort presented a wide range of distribution from grade 1 to grade 4 of the NCT-CTAE scale. Nevertheless, we could not prove an increased incidence of infectious adverse events in the lower vs. higher-level lymphopenia group. These data are in line with several studies presenting primarily mild to moderate infections during fingolimod (8, 18, 28) The most relevant infectious complication in fingolimod treated patients is defined by varicella-zoster virus (VZV) and herpes simplex virus (HSV) infection or reactivation (8, 18, 33). However, individual variation of VZV specific T cell responses are assumed to be more relevant for upcoming VZV activation rather than absolute lymphocyte counts (34, 35). Up to date, we are not able to confirm that monitoring of absolute lymphocyte count or its subtypes can assist to predict higher infectious risk at lymphocyte counts of 0.2 GPT/l. Instead, cases of severe upcoming disease activity after fingolimod cessation because of lymphopenia (< 0.2 GPT/l) are known (35, 36). Though, fingolimod interruption on the basis of lymphopenia of < 0.2 GPT/l has to be critically discussed in the individual context.

In summary, we demonstrate that CD4+ T cells and CD19+ B cells are comparably decreased in all fingolimod treated patients whereas grade of lymphopenia is primarily defined by individual variation of CD8+ T cell and NK cells. Monitoring of absolute lymphocyte drop and absolute lymphocyte count at steady state is only partially helpful as they do not cover distinct changes in the specific immune cell distribution. Our results suggest that monitoring of cellular target populations as CD4+ T cells seems to be more straight forward and probably clinically relevant to weigh and interpret the degree of immunological effects in fingolimod treated patients.

## Ethics statement

We confirm that any aspect of the work covered in this manuscript that has involved human patients has been conducted with the ethical approval of all relevant bodies (EK 348092014). The study was performed according the Declaration of Helsinki, and the study protocol was approved by the Ethics Committee of the Faculty of Medicine of the Dresden University of Technology. The authors have received consent forms from any participants in the study and have these forms available in case they are requested by the editor.

## Availability of the data and material

KA and TZ have full access to all the data in the study and take full responsibility for integrity of the data and the accuracy of the data analysis. Raw data are available on personal demand.

## Author contributions

KA and TZ: Study concept and design; MK: Acquisition of data; MK, KA, RH, and UP: Analysis and interpretation of data; MK, KA, and TZ: Drafting of the manuscript; UP and RH: Critical revision of the manuscript for important intellectual content; MK, and RH: Statistical analysis.

### Conflict of interest statement

KA received personal compensation for from Novartis, Biogen Idec, Roche, Sanofi, and Merck for consulting service. TZ received personal compensation from Biogen Idec, Bayer, Novartis, Sanofi, Teva, and Synthon for consulting services and received additional financial support for research activities from Bayer, Biogen Idec, Novartis, Teva, and Sanofi Aventis. UP speaker fee from Roche. RH received speaker fee from Sanofi. The remaining author declares that the research was conducted in the absence of any commercial or financial relationships that could be construed as a potential conflict of interest.

## Abbreviations

BMI: Body Mass Index
CNS: central nervous system
CRP: C-reactive protein
DMT: disease modifying drug
gamma-GT, EDSS: Expanded Disability Status Scale
EDTA: ethylene diamine tetra acetic acid
GLMM: Generalized Linear Mixed Model
MRI: magnet resonance imaging
MS: multiples sclerosis
NCI-CTCAE: National Cancer Institute Common Terminology Criteria for Adverse Events
NK cells: natural killer cells
RR: relapsing remitting
RWD: real-world data
RWE: real-world evidence
S1P: sphingosine-1-phosphate.
