# Supplementary material for: Real World Lab Data: Patterns of Lymphocyte Counts in Fingolimod Treated Patients
Source: Front Immunol. 2018 Nov 20;9:2669. doi: 10.3389/fimmu.2018.02669 (PMC6256977; doi:10.3389/fimmu.2018.02669)
Supplement: Supplementary file 1 [file Table_1.pdf]

Suppl. Table 1. Baseline characteristics

**Age (yr ± SD)**	39.5 (9.9)
**Duration since MS diagnosis (yr ± SD)**	6.7 (5.9)
**Sex (f/m)**	65/48
**Previous DMT use before fingolimod start (no. (%))**	91 (80.5)
**Interferon beta**	45 (39.8)
**Glatirameracetat**	27 (23.9)
**Natalizumab**	14 (12.4)
**Others**	5 (4.4)
**None previous DMT use (no. (%))**	22 (19.5)
**BMI (± SD)**	25.0 (4.7)
**EDSS (± SD)**	2.8 (1.4)

Baseline characteristics of evaluated patients are depicted. DMT, disease modifying treatment. Other previous treatments included: Azathioprin (2), Laquinimod (3). During a standardized treatment switch procedure, injectables were stopped 2 weeks before fingolimod start, natalizumab was stopped 12 weeks before fingolimod start and other DMTs at least 6 months before fingolimod start. Yr, year; SD, standard deviation; f, female; m, male; no, number.
